# Smoking and Its Effects

**Published:** 1861-02

**Authors:** 


Smoking and its Effects.—The pupils of the Polytechnic School in Paris, have recently furnished some curious statistics bearing on tobacco. Dividing the young gentlemen of that college into groups, the smokers and the non-smokers, it is shown that the smokers have proved themselves, in the various competitive examinations, far inferior to the others. Not only in the examinations on entering the school are the smokers in a lower rank, but in the various ordeals they have to pass through in a year, the average rank of the smokers had constantly fallen, and not inconsiderably, while the men that did not smoke enjoyed a cerebral atmosphere of the clearest kind. It would be interesting to pursue this kind of statistical inquiry in our public schools and universities. Perhaps smoking is, in many instances, not the cause, but the effect or indication of intellectual mediocrity. Is there any connection between smoking and German metaphysics?—*London Lancet.*

